# Effects of Dietary Metabolizable Energy and Crude Protein on Postprandial Metabolite Dynamics and Lactation Performance in Dairy Goats

**DOI:** 10.3390/metabo16070515

**Published:** 2026-07-22

**Authors:** Xiuqing Li, Lingbo Wang, Zhiyong Hu, Qiuling Hou, Yun Wang, Yizhao Shen, Yingyu Mu, Xueyan Lin, Zhonghua Wang

**Affiliations:** College of Animal Science and Technology, Shandong Agricultural University, Tai’an 271018, China

**Keywords:** Laoshan dairy goat, nitrogen utilization, amino acid profile, arterial plasma, milk composition

## Abstract

**Background:** Dietary metabolizable energy (ME) and crude protein (CP) levels are important for lactation performance and metabolic responses in dairy ruminants. This study aimed to evaluate the effects of dietary ME and CP levels on lactation performance and postprandial metabolic responses in lactating dairy goats. **Methods:** Goats were randomly assigned to a 4 × 4 two-factor Latin square experiment consisting of four 14 d periods. The dietary treatments were high energy, high protein (HEHCP); high energy, low protein (HELCP); low energy, high protein (LEHCP); and low energy, low protein (LELCP). Serial postprandial arterial blood samples were collected at 17 daytime time points to characterize temporal changes in plasma amino acids, biochemical parameters and hormones. **Results:** Increasing CP supply elevated milk yield (+6%) and lactose yield (+5%) but decreased milk fat yield (−8%; *p* ≤ 0.04). Increasing ME supply tended to enhance milk yield and milk fat yield and increased milk lactose content only under the high CP condition (ME × CP interaction: *p* = 0.04), suggesting that the response to ME supply depended partly on dietary CP level. High CP increased plasma branched chain amino acid concentrations, whereas high ME reduced Leu and Val under the high CP condition. Most plasma amino acids exhibited marked postprandial dynamics, decreasing initially and then stabilizing, with CP × time interactions observed for Leu, Met, and Phe (*p* ≤ 0.05). High ME decreased plasma AST activity and tended to reduce urea N concentration and also reduced ALT activity and increased glucose concentration under the high CP diet. Following morning feeding, plasma urea N showed a progressive postprandial decline (*p* = 0.02), with glucagon decreasing and both prolactin and growth hormone increasing, despite no dietary effects on mean plasma hormone concentrations. **Conclusions:** Overall, dietary ME and CP levels affected lactation performance and selected plasma metabolic indicators in lactating dairy goats. Coordinated energy and protein supply should be considered when formulating diets for lactating dairy goats. Serial postprandial sampling further revealed temporal changes in plasma metabolites and hormones, providing useful information for refining precision nutrition strategies during lactation.

## 1. Introduction

Global demand for goat milk and its products continues to grow. It is projected that global goat milk production will increase by over 30% by 2030 [1]. Goat milk is highly favored by consumers for its unique nutritional profile (e.g., more digestible protein and fat structures, rich in medium- and short-chain fatty acids, and potential low allergenicity), making it a premium health food [2,3]. However, milk yield varies substantially among dairy goat production systems, with reported values ranging from approximately 153 to 589 L per doe per year, indicating considerable potential for further productivity improvements [1]. Consequently, developing natural and effective nutritional strategies to enhance milk yield and quality has become a key focus for dairy producers, consumers, and researchers.

The production and composition of dairy products (such as fat, protein, and lactose content) are key indicators that determine their market value, processing characteristics, and nutritional quality. In ruminant nutrition, dietary energy (ME) and crude protein (CP) levels are the two most critical factors regulating milk production performance. Adequate energy supply is essential to meet the metabolic demands of milk protein, while dietary protein provides the necessary amino acids for milk protein synthesis [4,5,6]. Increasing energy supply can curvilinearly enhance milk production [7]. In addition to milk protein synthesis, dietary ME and CP supply may also influence milk fat and lactose production, and these responses can vary with energy source, CP level, and the balance between energy and protein supply [7,8,9,10]. Adequate energy supply can support milk yield and may influence lactose production through changes in glucogenic precursor supply and mammary glucose metabolism [11,12]. The effect of energy supply on milk fat can also be variable: greater availability of lipogenic substrates, especially acetate, generally favors mammary fatty acid synthesis and milk fat production [13], whereas adequate energy supply may maintain milk and milk-component yields under reduced protein supply without necessarily altering milk fat production [4].

Previous studies have confirmed that increasing energy intake improves nitrogen utilization by reducing plasma urea nitrogen and branched-chain amino acid (BCAA) levels, thereby enhancing amino acid uptake in peripheral tissues and its deposition in milk protein [8,9]. Rumen glucose infusion can increase mammary uptake of histidine (His), methionine (Met), and phenylalanine (Phe), reduce hepatic catabolism of these amino acids (AA), and enhance milk protein yield [11]. Similarly, increasing dietary CP or metabolizable protein supply from deficient to adequate levels generally increases milk yield and milk protein yield by increasing the supply of absorbable amino acids, whereas these responses may diminish when protein supply exceeds animal requirements [10,14]. In addition, increased CP supply may support lactose yield through changes in mammary glucose and amino acid utilization, whereas its effects on milk fat may vary with concurrent energy supply and mammary metabolic adaptation [7,10]. Therefore, ME and CP may differentially affect milk yield and major milk components through changes in glucose metabolism, nitrogen utilization, amino acid availability, and mammary nutrient supply [5,7,9,10,12].

Recent studies have shown that the effects of dietary energy and protein on milk protein synthesis in lactating animals extend beyond the simple regulation of AA distribution and utilization, involving multiple physiological mechanisms and regulatory processes. First, dietary energy and protein can influence milk protein synthesis by modulating circulating hormone concentrations [15,16]. Previous studies have demonstrated that growth hormone [17], insulin [18,19], glucagon [20,21], and prolactin (PRL) modulate milk protein synthesis to varying degrees [22]. Moreover, dietary intake influences lactation performance by altering postprandial serum metabolite levels. Recent evidence indicated that higher dietary protein intake early in the day enhanced milk fat yield and modulated energy metabolism through elevated plasma insulin and non-esterified fatty acid (NEFA) concentrations [16]. We hypothesized that dietary metabolizable energy (ME) and crude protein (CP) supplies would interactively affect lactation performance and postprandial plasma metabolic profiles in lactating dairy goats, and that adequate ME supply would enhance the productive and metabolic utilization of increased CP supply. Therefore, this study aimed to evaluate the effects of dietary ME and CP levels on milk production, milk composition, and plasma parameters (amino acids, metabolites, and hormones) in lactating Laoshan dairy goats. Serial postprandial blood sampling was employed to characterize temporal changes in plasma metabolic and endocrine profiles. This study may provide a basis for understanding postprandial metabolic dynamics during lactation and for evaluating dietary ME and CP effects in dairy goats.

## 2. Materials and Methods

### 2.1. Experimental Animal

Four second-parity Laoshan dairy goats, all of which had delivered twins in the current lactation, were used. The goats averaged 50 ± 10 days in milk (DIM) and 40 ± 2 kg body weight (BW). Surgery was performed on the goats about two weeks before the start of the experiment to elevate the right carotid artery to a subcutaneous level, thereby facilitating repeated carotid blood sampling at multiple time points. Goats were housed in individual pens throughout the experiment, including each 14 d experimental period, and were milked twice daily at 0800 and 1700 h. Experimental diets were fed daily at 0815 and 1740. Goats were allowed free access to water throughout the experiment. This study was conducted in accordance with the Guide for the Care and Use of Laboratory Animals and was approved by the Institutional Animal Care and Use Committee of Shandong Agricultural University (approval code: SDAUA-2021-302; date of approval: 2 March 2021).

### 2.2. Treatments and Experimental Design

Four Laoshan dairy goats were randomly assigned to a 4 × 4 two-factor Latin square experiment that consisted of four 14 d periods. Diets containing two levels of metabolizable energy (ME) [low energy (LE) and high energy (HE)] and two levels of crude protein (CP) [low crude protein (LCP) and high crude protein (HCP)] were used. The goats were randomly assigned within periods to one of four diets [(1) high energy, high protein (HEHCP; 18.2% CP, 12.39 MJ/kg); (2) high energy, low protein (HELCP; 16.7% CP, 12.47 MJ/kg); (3) low energy, high protein (LEHCP; 18.5% CP, 11.62 MJ/kg); and (4) low energy, low protein (LELCP: 16.5% CP, 11.56 MJ/kg)], and sample collection was conducted on the final day of each period. The experimental diets were formulated according to the Agricultural and Food Research Council (AFRC, 1993) recommendations [23] to meet mineral and vitamin requirements (Table 1).

### 2.3. Milk Sampling and Analysis

On the final day of each period, milk samples were collected from both the morning and evening milkings for subsequent composition analysis. Milk composition was analyzed using an infrared analyzer (Type 78110; FOSS, Hillerød, Denmark).

### 2.4. Blood Sampling and Analysis

Approximately two weeks before the start of the experiment, the right carotid artery of each goat was surgically transposed to a subcutaneous position to facilitate repeated arterial blood collection. No indwelling carotid catheter was used; therefore, catheter flushing or patency maintenance was not required. During each sampling day, blood samples were collected by trained veterinary personnel through superficial needle puncture of the subcutaneously transposed carotid artery according to a predetermined sampling timetable. The sampling team followed a synchronized clock and time reminders, and blood samples were collected within approximately ±2 min of each scheduled daytime sampling point. This approach avoided repeated deep arterial puncture and reduced the potential tissue trauma and stress associated with repeated arterial sampling. After each collection, local pressure was applied to ensure hemostasis, and the sampling site and general condition of each goat were monitored throughout the sampling period. All scheduled blood samples were successfully collected, and no samples were excluded because of sampling failure or abnormal animal condition. Feeding and milking times were standardized throughout the experiment, and blood collection was coordinated with these fixed management events. On the final day of each 14 d experimental period, blood samples were collected at 0800, 0830, 0900, 0930, 1000, 1030, 1100, 1130, 1230, 1330, 1430, 1530, 1730, 1800, 1830, 1900, and 1930 h, totaling 17 daytime sampling points. Plasma urea N, glucose, total protein (TP), alanine aminotransferase (ALT), aspartate aminotransferase (AST), albumin (ALB), and total cholesterol (TC) were measured using an automatic biochemical analyzer (Model 7020; Hitachi High-Technologies Corporation, Tokyo, Japan), according to the manufacturer’s instructions, with reference to previously reported measurements in lactating goats [24]. Insulin, glucagon, prolactin (PRL), and growth hormone (GH) were measured by radioimmunoassay (RIA) using commercial assay kits (Nanjing Ao Qing Biotechnology Co., Ltd., Nanjing, China), according to the manufacturer’s instructions and as described previously [24]. Amino acid (AA) concentrations were measured by liquid chromatography-tandem mass spectrometry (LC-MS/MS; Waters XEVO TQ-S Micro tandem quadrupole mass spectrometer, Waters Corporation, Milford, MA, USA), with reference to a previously described HPLC-MS/MS method for amino acid determination [25].

### 2.5. Statistical Analysis

Experimental data were analyzed using the PROC MIXED procedure in SAS version 9.2 for Windows. For milk yield, milk composition, and plasma variables averaged across all daytime sampling points, each goat × period combination was considered the experimental unit. The model included dietary ME level, dietary CP level, their interaction, and period as fixed effects, with goat included as a random effect. For evaluation of postprandial temporal dynamics, the original plasma data from all daytime sampling points were analyzed using a repeated measures mixed model. The goat × period combination was specified as the subject for repeated measurements, with sampling time treated as a repeated measure within each goat × period combination. The model included dietary ME level, dietary CP level, sampling time, all corresponding interactions among ME, CP, and sampling time, and period as fixed effects, with goat included as a random effect. First-order autoregressive [AR(1)] and spatial power [SP(POW)] covariance structures were compared to account for correlations among repeated measurements, and the covariance structure with the lowest Akaike Information Criterion (AIC) was selected for each variable. When significant effects were detected, means were separated using the PDIFF option with Tukey’s adjustment. Differences were considered significant at *p* ≤ 0.05, and 0.05 < *p* ≤ 0.10 was considered a tendency. Graphs were generated using GraphPad Prism (version 10.1.2; GraphPad Software).

## 3. Results

### 3.1. Lactation Performance

Increasing ME supply tended to increase milk yield and milk fat yield (both ~3% higher, *p* ≤ 0.08) but did not affect milk protein yield or content (*p* ≥ 0.14, Table 2). Increased ME supply increased lactose content only under the HCP treatment (ME × CP interaction: *p* = 0.04). Increasing CP supply resulted in a 6% increase in milk yield and a 5% increase in lactose yield (*p* ≤ 0.04) but reduced milk fat yield by 8% (*p* = 0.04). Concurrently, there was a tendency for milk yield and milk fat yield to be influenced by the ME × CP interaction (*p* ≤ 0.09).

### 3.2. Plasma Amino Acids

Among the essential amino acids (EAA), mean concentrations of methionine (Met), lysine (Lys), threonine (Thr), phenylalanine (Phe), histidine (His), tryptophan (Trp), and arginine (Arg) were unaffected by dietary metabolizable energy (ME) or crude protein (CP) supply (*p* ≥ 0.13, Table 3). Concentrations of Leu, Ile, and Val were higher with the HCP treatment than the LCP treatment (*p* ≤ 0.05). Increasing ME supply significantly reduced Leu and Val concentrations only under the HCP treatment (ME × CP interaction *p* ≤ 0.05) and tended to decrease Ile concentration only under the HCP treatment (ME × CP interaction: *p* = 0.07). Among nonessential amino acid (NEAA), concentrations of glycine (Gly), glutamic acid (Glu), glutamine (Gln), asparagine (Asn), aspartic acid (Asp), serine (Ser), and tyrosine (Tyr) were unaffected by ME or CP supply (*p* ≥ 0.11). Ala concentration increased with rising ME only under the HCP treatment (ME × CP interaction: *p* < 0.01). By contrast, increasing ME supply significantly increased cysteine (Cys) concentration only under the LCP treatment (ME × CP interaction: *p* < 0.01).

**Figure 1:**
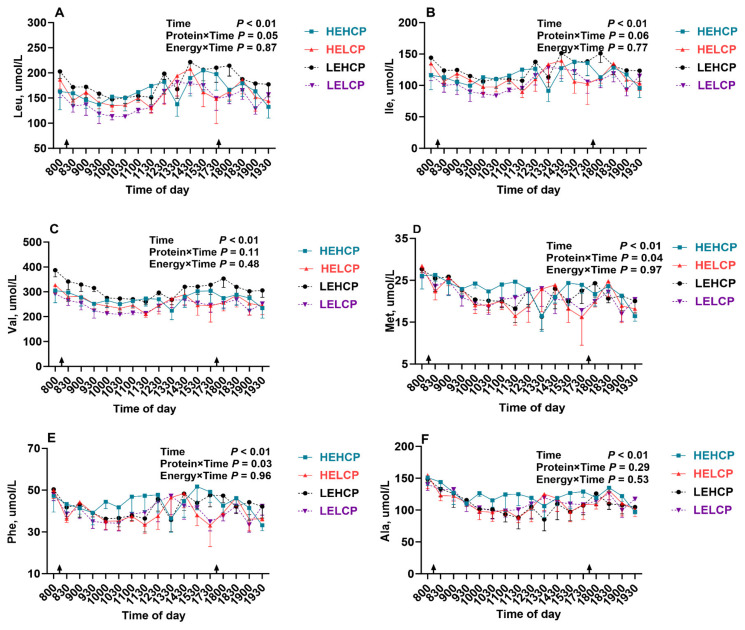
Postprandial changes in plasma amino acid concentrations in lactating dairy goats fed diets differing in energy and crude protein levels. (**A**) leucine (Leu); (**B**) isoleucine (Ile); (**C**) valine (Val); (**D**) methionine (Met); (**E**) phenylalanine (Phe); (**F**) alanine (Ala). Arrows indicate feeding times. Values are presented as means, with lower error bars representing the standard error of the mean (SEM). HEHCP, high energy and high crude protein; HELCP, high energy and low crude protein; LEHCP, low energy and high crude protein; LELCP, low energy and low crude protein. *p* ≤ 0.05 was considered significant, and 0.05 < *p* ≤ 0.10 was considered a trend.

Most plasma amino acid concentrations exhibited an initial decrease followed by stabilization during the postprandial period (Figure 1 and Figure S1). The main effect of sampling time was not significant for His, Glu, and Asp (*p* ≥ 0.27), whereas significant time effects were observed for the remaining amino acids (*p* ≤ 0.02). Arg, Ser, Gly, Thr, proline (Pro), Lys, and Met reached their lowest concentrations at 1130 h following morning feeding, with significant reductions compared with the concentrations at 0800 h (*p* ≤ 0.01). The magnitude of reduction varied among these amino acids, ranging from 24.52 ± 6.08 μmol/L for Ser to 186.48 ± 21.80 μmol/L for Gly. Furthermore, CP × time interactions were observed for Leu, Met, and Phe (*p* ≤ 0.05), whereas ME × time interactions were not significant (*p* ≥ 0.30).

### 3.3. Plasma Biochemical Parameters

**Figure 2:**
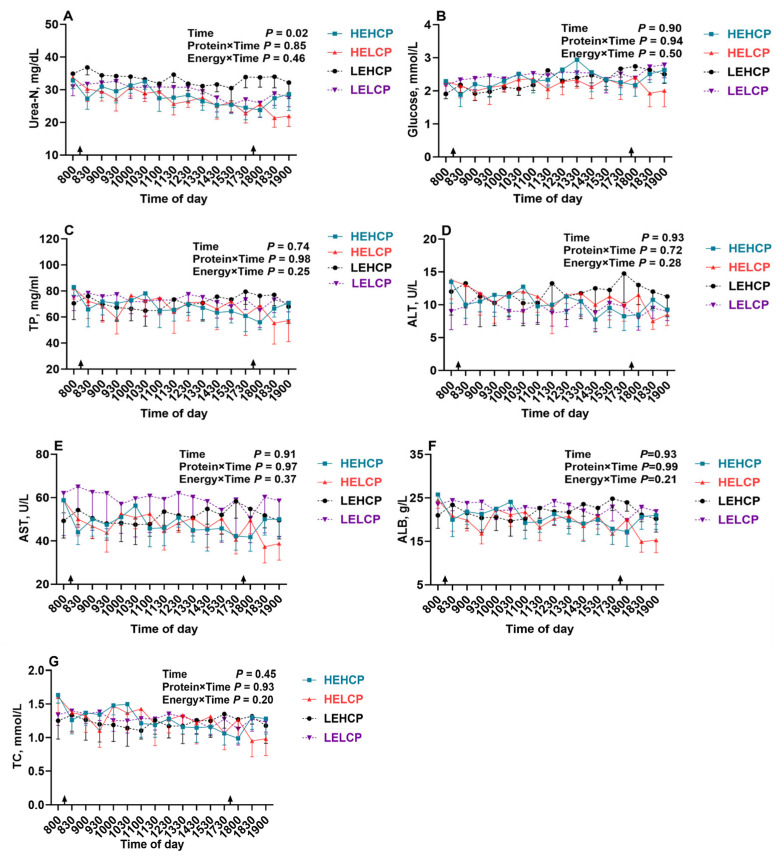
Postprandial changes in plasma biochemical parameters in lactating dairy goats fed diets differing in energy and crude protein levels. (**A**) Urea nitrogen (Urea N); (**B**) glucose; (**C**) total protein (TP); (**D**) alanine aminotransferase (ALT); (**E**) aspartate aminotransferase (AST); (**F**) albumin (ALB); (**G**) total cholesterol (TC). Values are presented as means, with lower error bars representing the standard error of the mean (SEM). *p* ≤ 0.05 was considered significant, and 0.05 < *p* ≤ 0.10 was considered a trend.

Compared with the LE treatment, the HE supply significantly decreased AST activity (*p* = 0.05) and showed a tendency to reduce arterial plasma urea N concentration and ALB concentration (*p* ≤ 0.09; Table 4). Furthermore, increased ME supply reduced plasma ALT or increased glucose concentration only under the HCP treatment (ME × CP interaction, *p* < 0.01). Urea N concentration increased with rising CP supply (*p* = 0.04), and this CP-induced increase tended to be greater under the LE diet than under the HE diet (ME × CP interaction, *p* = 0.06). Increased CP supply reduced glucose, ALB concentration, and AST activity only under the LE treatment (ME × CP interaction: *p* ≤ 0.09). TP and TC were unaffected by ME or CP supply (*p* ≥ 0.42). Plasma urea N concentration decreased over time (*p* = 0.02; Figure 2). In contrast, glucose, TP, ALT, AST, ALB, and TC concentrations showed no changes over time (*p* ≥ 0.45), and no treatment × time interaction effects were observed for any biochemical parameters (*p* ≥ 0.20).

### 3.4. Plasma Hormones

**Figure 3:**
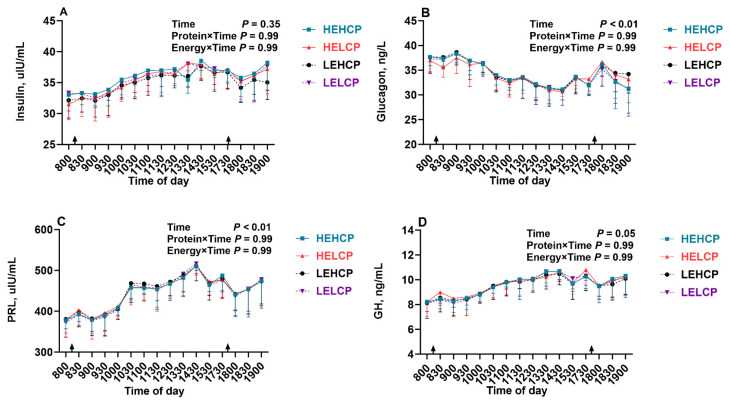
Postprandial changes in plasma hormone concentrations in lactating dairy goats fed diets differing in energy and crude protein levels. (**A**) insulin; (**B**) glucagon; (**C**) prolactin (PRL); (**D**) growth hormone (GH). Values are presented as means, with lower error bars representing the standard error of the mean (SEM). *p* ≤ 0.05 was considered significant, and 0.05 < *p* ≤ 0.10 was considered a trend.

Dietary ME and CP supply did not significantly affect the mean plasma concentrations of insulin, glucagon, PRL, or GH (*p* ≥ 0.18; Table 5). During the postprandial period, glucagon, PRL, and GH changed over time (*p* ≤ 0.05; Figure 3). Specifically, glucagon concentration decreased from 0800 to 1430 h following morning feeding, whereas PRL and GH concentrations increased over the same period. At 1430 h, PRL and GH concentrations tended to be higher than those at 0800 h, with increases of 135.13 ± 28.41 µIU/mL and 2.44 ± 0.74 ng/mL, respectively (*p* ≤ 0.09). No ME × time or CP × time interactions were observed for any hormone concentration (*p* = 0.99).

## 4. Discussion

Numerous studies have reported increased milk yield in response to elevated dietary protein and energy supply or post-ruminal infusions of glucose and AA [4,5,11,26]. Consistent with these findings, increasing CP and ME supply in the present study enhanced milk yield by 6% and 3%, respectively, although the effect of ME was only a trend. Notably, increasing CP supply under the HE treatment increased milk yield by 8.5% (155 g), compared with only 3.6% (67 g) under the LE treatment. These results suggest that the milk yield response to dietary protein was enhanced under the HE treatment, which is consistent with prior findings [10,11]. This phenomenon may be explained by protein × energy interactions at the animal level [7,27]. However, numerous studies involving post-ruminal infusions of amino acids and glucose or modifications to dietary protein and energy levels have reported independent additive effects of protein and energy on milk yield, without interaction [11,28,29]. In contrast, the study by Brun-Lafleur et al. [7], which involved a carefully controlled experiment with uniform distribution of energy and protein, revealed an interaction effect between protein and energy on lactation performance. Differences in experimental design, the range and distribution of dietary energy and protein levels, the route of nutrient supply, and animal or dietary conditions may partly explain the inconsistent detection of energy × protein interactions among studies. The observed interaction pattern was consistent with the accompanying metabolic changes. Under the HCP condition, increasing ME supply increased plasma glucose and reduced circulating Leu and Val concentrations, which may reflect altered amino acid partitioning and greater BCAA utilization or tissue uptake when energy supply is adequate [11,30]. The tendency toward lower urea N may also indicate reduced amino acid catabolism or improved nitrogen utilization. These changes may help explain the greater milk yield response to CP under HE than under LE, as well as the increase in lactose content when both ME and CP supplies were high. Given the modest number of animals, these interaction effects should be interpreted cautiously and confirmed in larger studies.

Multiple studies have demonstrated that dietary energy can shift AA towards mammary tissue, thereby increasing milk protein synthesis [9,31]. However, our results did not support this observation. Although increasing net energy intake for lactation or administering post-ruminal glucose infusions could enhance milk protein yield or concentration under the certain conditions [9,31], other studies have found no significant effect [32,33]. For example, Nichols et al. [32] reported that supplementing glucose during essential amino acid infusions enhances the transfer of BCAAs to peripheral tissues, thereby reducing plasma BCAA concentrations. Consequently, milk protein yield did not increase, indicating that the redirection of BCAAs to peripheral tissues could limit the ability of glucose to enhance milk protein synthesis. Based on these findings, we hypothesize that elevated plasma amino acid levels may attenuate glucose’s stimulatory effect on milk protein yield by promoting amino acid storage in extra-mammary tissues, thus limiting the potential benefits of increased dietary energy for milk protein synthesis. Moreover, we found that increasing dietary ME supply tended to increase milk fat yield, which aligns with the effects of post-ruminal glucose infusions or starch supply [29,34]. These studies indicate that milk fat content and yield are higher when the supply of gluconeogenic precursors is low, whereas acetate supplementation experiments increased milk fat yield [13,35]. These findings suggest that changes in dietary energy supply may alter nutrient partitioning between glucose related metabolism and milk fat synthesis. We further observed that elevated dietary CP supply reduced milk fat yield whilst increasing lactose yield. Lactose and milk fat yields showed no increase in some studies [5], but increased concurrently in others [9,10]. In J. C. Anger et al.’s study, elevated dietary CP supply showed an upward trend in mammary glucose uptake (MGU) [10], thereby enhancing lactose production; as amino acid supply increased, systemic glucose utilization correspondingly improved [12]. Synthesizing these findings, we hypothesize that elevated dietary CP levels in the present study may have enhanced mammary glucose uptake and utilization, thereby promoting lactose synthesis. From a practical perspective, these findings suggest that dietary ME and CP supplies should be balanced according to the production targets of lactating dairy goats. Increasing CP supply may improve milk yield and lactose yield, as observed in the present study. Adequate energy supply is important for the efficient utilization of absorbed amino acids and for supporting the productive response to dietary protein [4,9]. Increasing CP beyond productive requirements may reduce nitrogen-use efficiency and increase urea N and urinary N excretion when the additional protein does not result in a corresponding improvement in milk production [36]. Therefore, ration formulation should consider lactation stage, milk yield, forage quality, and the desired milk component profile rather than increasing dietary CP alone. Plasma or milk urea N may provide useful supporting information when evaluating dietary protein supply and nitrogen-use efficiency, although sampling time should be standardized [36].

In agreement with previous findings [4], the concentrations of the BCAAs of Leu, Ile, and Val changed in response to variations in dietary energy and protein levels. This phenomenon is likely due to the different catabolic patterns of BCAAs and other EAAs across various tissues. The first step in BCAA catabolism is catalyzed by branched-chain amino acid transaminase (BCAT), which exhibits high activity in peripheral tissues such as skeletal muscle, heart, and adipose tissue but is minimally expressed in the liver [37]. As a result, BCAAs are minimally catabolized in the liver, whereas other amino acids are predominantly catabolized there. For example, modeling by Fleming et al. predicts hepatic absorption and utilization rates of 34–55% for Arg, His, Met, and Phe, whereas BCAA utilization rates remain below 4% [38]. As a result, changes in plasma BCAA concentrations due to increased dietary protein levels are more evident in circulating concentrations [4]. Increasing CP supply increased plasma Leu, Ile, and Val concentrations, and this was accompanied by higher milk yield and lactose yield. This result is broadly consistent with infusion studies in lactating dairy cows showing that increased EAA supply elevated arterial BCAA concentrations and mammary EAA uptake, together with milk yield and lactose responses [32]. From the perspective of milk chemical composition, BCAAs are important constituents of milk protein, and Leu can regulate casein expression through the mTOR related amino acid sensing pathway [39]. In addition, lactose is the major osmotic component of milk and an important determinant of milk volume [40]. However, HCP reduced milk fat yield and did not significantly increase milk protein yield, indicating that higher circulating BCAA availability was associated mainly with milk yield and lactose yield, without coordinated increases in all milk components. Similarly, Curtis et al. reported that BCAA supplementation during glucose infusion increased arterial BCAA concentrations but did not improve milk component synthesis consistently, likely because amino acids were redirected toward extra mammary tissues [31].

Numerous studies have shown that increased dietary energy supply can reduce blood BCAA concentrations [31,32]. We observed that increasing energy supply under the HCP treatment conditions alone reduced plasma BCAA concentrations. This phenomenon may be related to the elevated circulating glucose concentrations accompanying increased energy levels under the HCP conditions. Cant et al. found that glucose enhances the net plasma clearance of BCAAs by mammary and extramammary tissues, thus reducing their circulating levels [30]. Additionally, Nichols et al. indicated that when EAAs do not limit milk protein synthesis, elevated blood glucose concentrations redirect amino acids to peripheral tissues, promoting protein synthesis there [32]. This may provide a possible explanation for the effect of dietary energy on plasma BCAA concentrations. Furthermore, the reduction in BCAA levels is unlikely to be due to increased BCAA catabolism, as plasma Urea-N concentrations decreased by 3.72 mg/dL in the HEHCP group compared to the LEHCP group. In addition, increasing ME supply under the HCP condition increased milk lactose content. This response was partly consistent with the findings of Omphalius et al. [9], who reported that greater energy supply reduced circulating BCAA concentrations and increased milk protein yield, milk protein content, and lactose content in dairy cows. However, unlike their study, milk protein content and milk protein yield were not affected by ME supply in the present study. These results suggest that, under the HCP condition, the effect of ME may be more closely associated with glucose availability and lactose synthesis than with measurable increases in gross milk protein output. Therefore, changes in plasma BCAA concentrations may reflect systemic amino acid partitioning and tissue utilization status, and further studies measuring milk amino acid composition, milk fatty acid composition, mammary nutrient uptake, and BCAA catabolism are needed to determine their effects on the detailed nutritional quality of goat milk.

Among the NEAAs, only the plasma concentrations of Ala and Cys were influenced by ME and CP supply. Ala is a glucogenic AA and contributes to gluconeogenesis [41]. In the present study, the HCP treatment provided abundant glucogenic AA, particularly Ala. When energy is insufficient, the body utilizes Ala for gluconeogenesis, thus reducing plasma Ala concentration. The highest plasma Urea-N concentration in the LEHCP group also indicates enhanced amino acid catabolism. In contrast, under the HEHCP condition, Ala does not need to be catabolized for energy. This explains why plasma Ala concentrations were lowest in the LEHCP group and highest in the HEHCP group in the present study. Notably, the HEHCP group also showed a relatively high milk lactose content. Because glucose is the primary precursor for lactose synthesis [42], the increase in plasma Ala under the HEHCP condition may be associated with greater glucogenic precursor availability and the higher milk lactose content observed in this group. Cys is a sulfur-containing amino acid. Met is first converted to S-adenosylmethionine (SAM) with ATP as the energy source, then to Cys via the transsulfuration pathway [43]. In the HELCP diet, increased energy availability may have promoted the conversion of Met to Cys, thereby contributing to higher plasma Cys concentrations. In addition, abundant energy supply may reduce the need for Cys catabolism, helping to preserve circulating Cys. From the perspective of milk nutritional quality, changes in Cys availability may influence the supply of sulfur-containing amino acids for milk protein synthesis, potentially through the utilization of circulating glutathione by the mammary gland [44]. However, milk amino acid composition, milk protein fractions, glutathione concentration, and antioxidant components were not measured in the present study; therefore, further studies are needed to confirm whether these NEAA changes affect the detailed nutritional quality of goat milk.

Fluctuations in dietary nutrients can affect blood metabolites involved in nutrient metabolism. These metabolites may act as metabolic signals that regulate lactation performance and metabolic health in dairy animals. Nitrogen efficiency has been evaluated at varying levels of CP or metabolizable protein. These studies aimed to assess the response to changes in energy status resulting from modifications in dietary energy levels [4,9,36] or post-ruminal glucose infusion [11]. Plasma urea N is commonly used as an indicator of protein and nitrogen metabolic status in ruminants because it reflects the balance among dietary nitrogen supply, amino acid catabolism, and nitrogen utilization [45]. This is consistent with prior findings from studies on increased protein intake [4,9], where increased protein intake results in relatively higher plasma Urea-N levels, regardless of dietary energy levels. Conversely, Urea-N concentration decreases as dietary energy levels increase, reaching their minimum in HELCP diets. Therefore, reducing dietary protein intake while appropriately increasing energy supply is crucial for improving nitrogen utilization efficiency and mitigating environmental pollution.

In the present study, arterial glucose concentrations increased only under the HCP conditions as ME supply increased, with the highest levels observed in the LELCP diet group. This contrasts with previous findings that increased dietary protein levels do not affect plasma glucose concentrations, whereas elevated dietary energy supply elevates plasma glucose level [4,29]. Serum glucose is an important indicator of energy metabolism in dairy animals. Insufficient exogenous energy and protein supply triggers the mobilization of body fat and muscle tissue, leading to elevated blood concentrations of NEFA and gluconeogenic precursors. NEFA serve as crucial substrates for milk fat synthesis [46]. The LELCP group exhibited a relatively high milk fat yield, suggesting the possibility of enhanced endogenous energy mobilization under the LELCP condition, which may also have contributed to the numerically higher glucose concentration. Plasma ALT, AST, and TC are regarded as biomarkers reflecting hepatic function. ALT and AST play crucial roles in hepatic amino acid metabolism and gluconeogenesis. In the present study, elevated ME levels reduced plasma AST and decreased ALT activity only under the HCP treatment, potentially indicating that dietary energy supplementation alleviates hepatic dysfunction.

In lactating mammals, the effect of dietary nutrient levels on plasma hormones is a complex, multifactorially regulated process. Dietary protein levels and energy supply exert no influence on insulin, glucagon, or GH, which is broadly consistent with previous reports of limited changes in plasma GH or insulin in response to dietary protein or carbohydrate treatments [47,48]. This indicates that, at the population level, protein itself is not an independent driver of these three hormones. It is commonly assumed that changes in plasma glucose concentration induced by dietary energy supply in ruminants are accompanied by alterations in plasma insulin concentrations. However, this phenomenon was not observed in the present trial, consistent with results from duodenal glucose infusion or prepartum feeding of high-energy diets [12,49]. Conversely, Piccioli-Cappelli et al. found that only increasing dietary starch content during early lactation concurrently elevated plasma glucose and insulin concentrations [34], with insulin’s response to glucose supply being nutritionally dependent [50]. Reducing dietary energy levels reduces energy intake and impairs energy balance, though this effect may be insufficient to alter hormone concentrations. Additionally, individual animal variation and baseline plasma glucose concentrations likely represent significant contributing factors. Whilst glucagon secretion is stimulated by hypoglycemia, plasma glucose concentrations across the dietary gradient used in this trial may not have fallen sufficiently to induce a significant increase in glucagon secretion. In the present trial, prolactin remained unaffected by dietary energy and protein supply, consistent with findings from studies feeding lactating cows diets with varying energy and protein levels during the dry period [15]. This response may reflect the role of prolactin in maintaining steady-state lactation.

Most plasma amino acid concentrations and several hormones changed over time after morning feeding, whereas among the biochemical parameters, only plasma urea N showed a significant temporal change. Consistent with previous findings [34], this study observed a gradual decrease in plasma amino acid concentrations over time, a phenomenon potentially linked to dynamic hormonal changes. Specifically, reduced glucagon levels may diminish amino acid catabolism and stimulate protein synthesis [51]. Furthermore, prolactin may further reduce postprandial plasma amino acid concentrations by promoting mammary cell proliferation and milk protein synthesis [52,53]. Together, these temporal patterns suggest that hormonal changes may be associated with postprandial amino acid utilization during lactation. These temporal endocrine and metabolic patterns may indicate a temporary shift toward nutrient utilization for lactation after feeding. The temporal changes in hormones, amino acids, and urea N also suggest that sampling time should be standardized when these indicators are used to evaluate nutritional status. In addition, evidence from dairy cows indicates that protein supply timing can affect daily rhythms of milk synthesis, plasma hormones, and metabolites [16]. From a practical perspective, diets for lactating goats should be formulated by considering ME and CP supply together, while metabolic indicators should be sampled at a consistent interval relative to feeding. These practices may improve the consistency of nutritional assessment and assist the interpretation of milk-yield and milk-component response. However, these practical implications should be further validated in larger herds and longer-term feeding trials.

Although the 4 × 4 Latin square design allowed each goat to receive all dietary treatments and reduced between-animal variation, the small number of goats limited statistical power and may restrict the generalizability of the findings. Therefore, the observed treatment responses, particularly tendency-level effects and interaction patterns, should be interpreted with caution and confirmed in larger studies.

## 5. Conclusions

Dietary ME and CP levels jointly influenced lactation performance and postprandial metabolic status in lactating Laoshan dairy goats. Increasing CP level increased milk yield and lactose yield but decreased milk fat yield, indicating that CP altered milk component partitioning. Increasing ME level tended to increase milk production and modified amino acid and metabolic responses mainly under the high CP condition, suggesting that the effect of energy level was partly dependent on protein level. The pronounced postprandial dynamics of plasma amino acids, urea N, and hormones indicate that single-time-point sampling may not fully capture postprandial metabolic changes during lactation. These findings highlight the importance of considering dietary nutrient balance together with postprandial temporal dynamics in lactation studies. In practical diet formulation for lactating dairy goats, coordinated energy and protein supply should also be considered.

## Figures and Tables

**Table 1:** Ingredients and nutrients of the diet.

Item ^1^	Diet ^2^			
	HEHCP	HELCP	LEHCP	LELCP
Ingredient, % of DM				
Corn	10	16	1.3	7.3
Soybean meal	25.5	19.5	25.5	19.5
Bran	2.5	2.5	2.5	2.5
Peanut vine	29.8	29.8	38.5	38.5
Alfalfa Hay	29.7	29.7	29.7	29.7
CaHPO_4_	0.1	0.1	0.1	0.1
CaCO_3_	0.9	0.9	0.9	0.9
Salt	0.5	0.5	0.5	0.5
Premix ^3^	1	1	1	1
Total	100	100	100	100
Nutrients				
DM, % of Diet	89.46	88.92	89.07	87.80
CP, % of DM	18.2	16.7	18.5	16.5
NDF, % of DM	28.73	32.70	28.55	30.09
ADF, % of DM	21.88	23.52	22.99	24.88
Ash, % of DM	10.06	10.57	9.62	11.53
AIA, % of DM	4.46	4.60	4.21	4.92
EE, % of DM	1.32	1.39	1.34	1.41
Ca, % of DM	1.91	2.03	1.79	2.18
P, % of DM	1.42	1.54	1.37	1.56
ME, MJ/kg of DM ^4^	12.39	12.47	11.62	11.56

^1^ DM, dry matter; CP, crude protein; NDF, neutral detergent fiber; ADF, acid detergent fiber; AIA, acid-insoluble ash; EE, ether extract; Ca, calcium; P, phosphorus; ME, metabolizable energy. ^2^ HEHCP = high energy, high crude protein; HELCP = high energy, low crude protein; LEHCP = low energy, high crude protein; LELCP = low energy, low crude protein; ^3^ The premix contains Copper sulfate (CuSO_4_), Manganese sulfate (MnSO_4_), Zinc sulfate (ZnSO_4_), Cobalt chloride (CoCl_2_), Iodate, Disodium selenite, Vitamin A, Vitamin D, and Vitamin E. ^4^ ME values were calculated based on diet composition and nutrient values using the AFRC (1993) system [23]. HE and LE denote the designed high- and low-ME treatments, respectively.

**Table 2:** Effect of ME and CP supplies on milk yield and milk composition in dairy goats.

	Diet ^1^				SEM	*p*-Value ^2^		
Item	HEHCP	HELCP	LEHCP	LELCP		Energy	Protein	ME × CP
Milk yield, g/d	1980 ^a^	1825 ^b^	1889 ^a^	1822 ^b^	224.03	0.06	0.04	0.07
Milk fat, %	2.65	2.83	2.46	3.04	0.51	0.35	0.31	0.35
Milk fat yield, g/d	54.28 ^b^	57.74 ^a^	51.48 ^b^	57.31 ^a^	12.14	0.08	0.04	0.09
Milk protein, %	2.65	2.83	2.62	2.81	0.08	0.50	0.44	0.45
Milk protein yield, g/d	54.71	56.64	54.41	53.50	6.76	0.14	0.06	0.15
Lactose, %	4.18 ^b^	4.11 ^d^	4.16 ^c^	4.21 ^a^	0.10	0.03	0.03	0.04
Lactose yield, g/d	84.66 ^a^	82.13 ^b^	86.48 ^a^	80.15 ^b^	8.89	0.11	0.02	0.05
Solids, %	10.27	10.50	10.00	10.78	0.62	0.54	0.50	0.55
SNF, % ^3^	7.53	7.64	7.45	7.68	0.15	0.45	0.40	0.45
Urea-N, mg/dL	22.96	20.85	22.76	21.91	1.51	0.24	0.23	0.23

^1^ HEHCP = high energy, high crude protein; HELCP = high energy, low crude protein; LEHCP = low energy, high crude protein; LELCP = low energy, low crude protein. ^2^ *p*-Values correspond to the effect of energy supply (LE vs. HE), effect of protein supply (LCP vs. HCP), and interaction between energy and protein (ME × CP). ^a–d^ Means within a row with no common superscripts differ (*p* ≤ 0.05). Trends were declared at 0.05 < *p* ≤ 0.10. ^3^ SNF = solids-not-fat.

**Table 3:** Effects of ME and CP supplies on mean plasma amino acid concentrations in dairy goats.

Item ^1^	Diet ^2^				SEM	*p*-Value ^3^		
	HEHCP	HELCP	LEHCP	LELCP		Energy	Protein	ME × CP
EAA								
Leu	164.59 ^b^	158.87 ^c^	180.62 ^a^	146.30 ^d^	10.45	0.05	0.03	0.05
Met	22.77	20.93	21.84	21.19	1.92	0.13	0.14	0.13
Lys	120.91	117.58	125.81	117.24	21.77	0.76	0.72	0.75
Ile	115.99 ^b^	112.54 ^c^	126.43 ^a^	106.10 ^d^	8.64	0.07	0.05	0.07
Val	272.94 ^b^	259.49 ^c^	309.86 ^a^	245.31 ^d^	15.30	0.01	<0.01	<0.01
Thr	70.42	64.85	72.42	66.51	4.54	0.68	0.76	0.70
Phe	43.81	39.56	42.28	39.81	2.88	0.16	0.18	0.15
His	45.82	47.10	44.71	42.83	2.56	0.15	0.19	0.19
Arg	185.27	181.47	183.16	171.36	17.91	0.69	0.70	0.73
Trp	45.29	41.96	43.75	41.09	3.18	0.29	0.31	0.27
NEAA								
Ala	123.55 ^a^	111.70 ^b^	109.54 ^c^	111.78 ^b^	9.76	<0.01	<0.01	<0.01
Gly	469.47	452.88	413.45	415.54	49.34	0.17	0.11	0.11
Glu	37.65	37.38	36.49	37.14	2.22	0.50	0.46	0.47
Gln	234.53	229.30	218.99	219.09	10.72	0.38	0.32	0.31
Asn	63.87	58.08	61.30	55.69	6.29	0.54	0.55	0.50
Asp	2.69	2.92	2.80	2.54	0.36	0.33	0.34	0.35
Ser	71.58	68.59	69.29	62.31	9.95	0.73	0.75	0.81
Pro	121.81	117.33	126.89	103.63	14.84	0.08	0.07	0.09
Cys	24.53 ^b^	27.01 ^a^	24.79 ^b^	24.46 ^b^	3.01	<0.01	<0.01	<0.01
Tyr	56.40	53.98	57.15	49.79	3.72	0.45	0.42	0.48

^1^ EAA, essential amino acids; NEAA, nonessential amino acids; Leu, leucine; Met, methionine; Lys, lysine; Ile, isoleucine; Val, valine; Thr, threonine; Phe, phenylalanine; His, histidine; Arg, arginine; Trp, tryptophan; Ala, alanine; Gly, glycine; Glu, glutamate; Gln, glutamine; Asn, asparagine; Asp, aspartic acid; Ser, serine; Pro, proline; Cys, cysteine; Tyr, tyrosine. ^2^ HEHCP = high energy, high crude protein; HELCP = high energy, low crude protein; LEHCP = low energy, high crude protein; LELCP = low energy, low crude protein. ^3^ ME = metabolizable energy; CP = crude protein; SEM = standard error of the mean. *p*-Values correspond to the effect of energy supply (LE vs. HE), effect of protein supply (LCP vs. HCP), and interaction between energy and protein (ME × CP). ^a–d^ Means within a row with no common superscripts differ (*p* ≤ 0.05). Trends were declared at 0.05 < *p* ≤ 0.10.

**Table 4:** Effects of ME and CP supplies on mean concentrations of plasma metabolites in dairy goats.

Item ^1^	Diet ^2^				SEM	*p*-Value ^3^		
	HEHCP	HELCP	LEHCP	LELCP		Energy	Protein	ME × CP
Urea N, mg/dL	29.59 ^a^	27.01 ^b^	33.31 ^a^	28.09 ^b^	0.86	0.09	0.04	0.06
Glucose, mmol/L	2.37 ^b^	2.19 ^d^	2.31 ^c^	2.47 ^a^	0.24	<0.01	<0.01	<0.01
TP, mg/mL	68.20	68.30	71.50	73.20	9.60	0.74	0.83	0.84
ALT, U/L	10.31 ^c^	10.95 ^b^	11.94 ^a^	9.52 ^d^	2.13	<0.01	<0.01	<0.01
AST, U/L	48.10 ^b^	47.50 ^b^	51.40 ^a^	59.40 ^a^	7.68	0.05	0.07	0.08
ALB, g/L	20.70	19.50	21.80	22.70	1.96	0.06	0.09	0.09
TC, mmol/L	1.28	1.27	1.23	1.28	0.17	0.45	0.42	0.44

^1^ Urea N = Urea nitrogen; TP = total protein; ALT = alanine aminotransferase; AST = aspartate aminotransferase; ALB = albumin; TC = total cholesterol. ^2^ HEHCP = high energy, high crude protein; HELCP = high energy, low crude protein; LEHCP = low energy, high crude protein; LELCP = low energy, low crude protein. ^3^ *p*-Values correspond to the effect of energy supply (LE vs. HE), effect of protein supply (LCP vs. HCP), and interaction between energy and protein (ME × CP). ^a–d^ Means within a row with no common superscripts differ (*p* ≤ 0.05). Trends were declared at 0.05 < *p* ≤ 0.10.

**Table 5:** Effects of ME and CP supplies on mean concentrations of plasma hormones in dairy goats.

Item ^1^	Diet ^2^				SEM	*p*-Value ^3^		
	HEHCP	HELCP	LEHCP	LELCP		Energy	Protein	ME × CP
Insulin, μIU/mL	35.90	35.44	34.94	35.65	2.07	0.20	0.18	0.19
Glucagon, ng/L	34.22	34.12	34.47	34.06	1.91	0.86	0.84	0.86
PRL, μIU/mL	443.92	445.00	446.60	443.85	22.19	0.84	0.84	0.84
GH, ng/mL	9.54	9.59	9.47	9.52	0.74	0.97	0.99	0.98

^1^ PRL = prolactin; GH = growth hormone. ^2^ HEHCP = high energy, high crude protein; HELCP = high energy, low crude protein; LEHCP = low energy, high crude protein; LELCP = low energy, low crude protein. ^3^ *p*-Values correspond to the effect of energy supply (LE vs. HE), effect of protein supply (LCP vs. HCP), and interaction between energy and protein (ME × CP). Differences were considered significant at *p* ≤ 0.05, and trends were declared at 0.05 < *p* ≤ 0.10.

## Data Availability

The original contributions presented in this study are included in the article. Further inquiries can be directed to the corresponding authors.

## Supplementary Materials

The following supporting information can be downloaded at https://www.mdpi.com/article/10.3390/metabo16070515/s1, Figure S1: Postprandial changes in the remaining plasma amino acid concentrations in lactating dairy goats fed diets differing in energy and crude protein levels. (A) Lysine; (B) Threonine; (C) Histidine; (D) Arginine; (E) Tryptophan; (F) Glycine; (G) Glutamic acid; (H) Glutamine; (I) Asparagine; (J) Aspartic acid; (K) Serine; (L) Proline; (M) Cysteine; (N) Tyrosine. Arrows indicate feeding times. Values are presented as means, with lower error bars representing the standard error of the mean (SEM). *p* ≤ 0.05 was considered significant, and 0.05 < *p* ≤ 0.10 was considered a trend.

## Abbreviations

The following abbreviations are used in this manuscript:

AA	Amino acid
ALB	Albumin
ALT	Alanine aminotransferase
AST	Aspartate aminotransferase
BCAA	Branched-chain amino acid
BW	Body weight
CP	Crude protein
DIM	Days in milk
EAA	Essential amino acid
GH	Growth hormone
HE	High energy
HCP	High crude protein
HEHCP	High energy and high crude protein
HELCP	High energy and low crude protein
LC-MS/MS	Liquid chromatography-tandem mass spectrometry
LE	Low energy
LCP	Low crude protein
LEHCP	Low energy and high crude protein
LELCP	Low energy and low crude protein
ME	Metabolizable energy
NEAA	Nonessential amino acid
NEFA	Non-esterified fatty acid
PRL	Prolactin
RIA	Radioimmunoassay
TC	Total cholesterol
TP	Total protein
Urea N	Urea nitrogen
