# Supplementary material for: Effects of Dietary Metabolizable Energy and Crude Protein on Postprandial Metabolite Dynamics and Lactation Performance in Dairy Goats
Source: Metabolites. 2026 Jul 22;16(7):515. doi: 10.3390/metabo16070515 (PMC13414008; doi:10.3390/metabo16070515)
Supplement: Supplementary file 1 [file metabolites-16-00515-s001.zip › metabolites-4381393-supplementary.pdf]

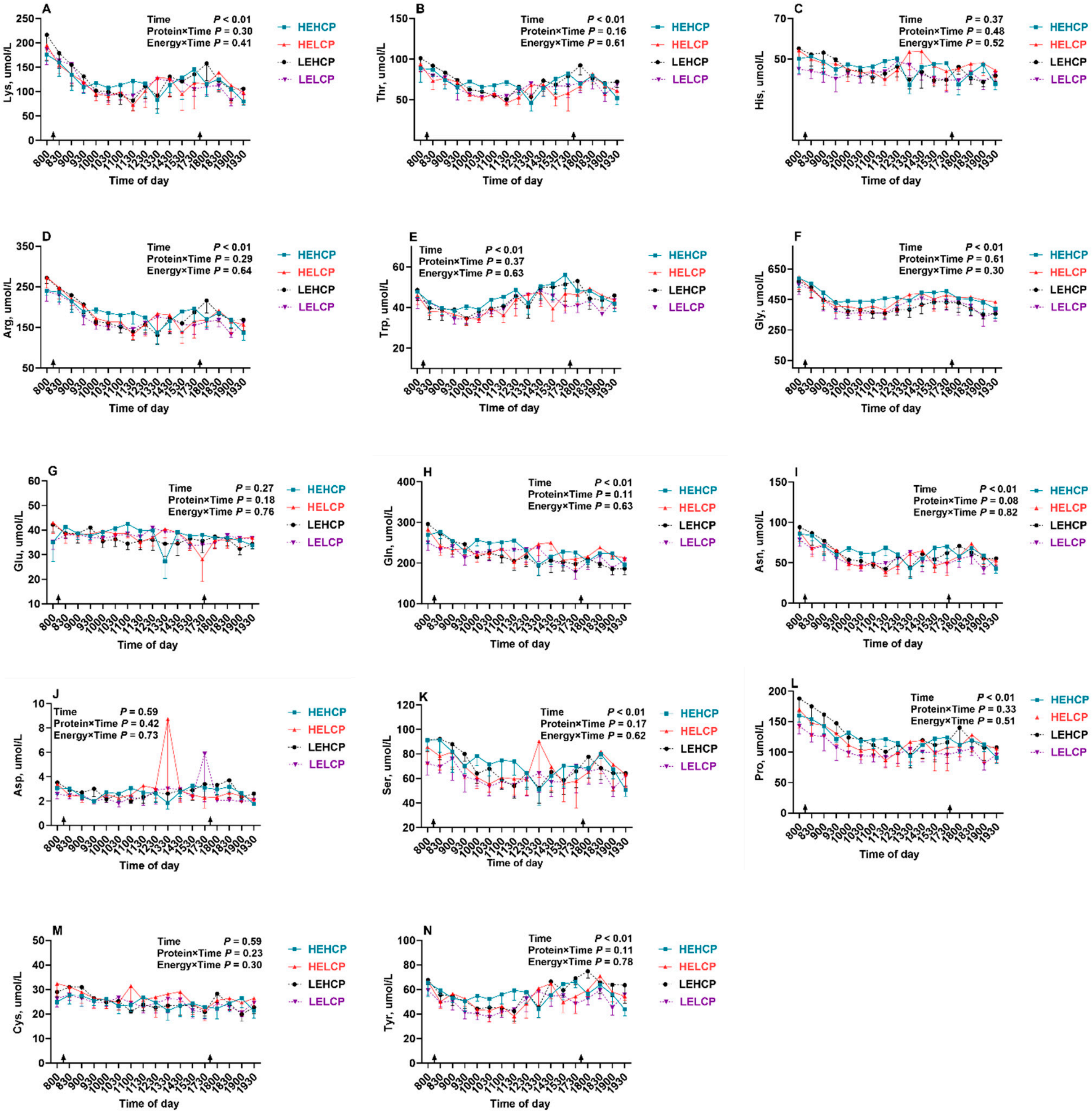

Figure S1. Postprandial changes in the remaining plasma amino acid concentrations in lactating dairy goats fed diets differing in energy and crude protein levels. (**A**) Lysine; (**B**) Threonine; (**C**) Histidine; (**D**) Arginine; (**E**) Tryptophan; (**F**) Glycine; (**G**) Glutamic acid; (**H**) Glutamine; (**I**) Asparagine; (**J**) Aspartic acid; (**K**) Serine; (**L**) Proline; (**M**) Cysteine; (**N**) Tyrosine. Arrows indicate feeding times. Values are presented as means, with lower error bars representing the standard error of the mean (SEM). $p \leq 0.05$ was considered significant, and $0.05 < p \leq 0.10$ was considered a trend.
